# Stress and the domestic cat: have humans accidentally created an animal mimic of neurodegeneration?

**DOI:** 10.3389/fneur.2024.1429184

**Published:** 2024-07-19

**Authors:** Ingrid R. Niesman

**Affiliations:** Department of Biology, SDSU Electron Microscopy Facility, San Diego State University, San Diego, CA, United States

**Keywords:** neurodegeneration, fCDS, ER stress, oxidative stress, neuroinflammation

## Abstract

Many neurodegenerative diseases (NDD) appear to share commonality of origin, chronic ER stress. The endoplasmic reticulum (ER) is a dynamic organelle, functioning as a major site of protein synthesis and protein posttranslational modifications, required for proper folding. ER stress can occur because of external stimuli, such as oxidative stress or neuroinflammatory cytokines, creating the ER luminal environment permissive for the accumulation of aggregated and misfolded proteins. Unresolvable ER stress upregulates a highly conserved pathway, the unfolded protein response (UPR). Maladaptive chronic activation of UPR components leads to apoptotic neuronal death. In addition to other factors, physiological responses to stressors are emerging as a significant risk factor in the etiology and pathogenesis of NDD. Owned cats share a common environment with people, being exposed to many of the same stressors as people and additional pressures due to their “quasi” domesticated status. Feline Cognitive Dysfunction Syndrome (fCDS) presents many of the same disease hallmarks as human NDD. The prevalence of fCDS is rapidly increasing as more people welcome cats as companions. Barely recognized 20 years ago, veterinarians and scientists are in infancy stages in understanding what is a very complex disease. This review will describe how cats may represent an unexplored animal mimetic phenotype for human NDD with stressors as potential triggering mechanisms. We will consider how multiple variations of stressful events over the short-life span of a cat could affect neuronal loss or glial dysfunction and ultimately tip the balance towards dementia.

## Introduction

Despite the original hypothesis proposed by Chambers et al. (1), the domestic cat is an underutilized model for NDD. When compared to transgenic rodent models or primates (2), felines are the only species extensively studied in which β-amyloid plaques (Aβ) and neurofibrillary hyperphosphorylated tau tangles (NFT) are found naturally comorbid (1, 3–6). Finding a translational animal model where early dementia-like behaviors and correlative biochemical signatures can predict final cognitive decline is a big leap forward in our understanding of the etiology of these devastating diseases. Cats, with significantly shorter life spans than humans, can bridge our gap in understanding the temporal progression in NDD and in our current futility in finding clinically efficacious therapies that go beyond simple temporary cognitive gains.

Modern human and animal medicine has greatly lengthened lifespans with a staggering increase in reported cognitive issues and dementia in people and cats alike. There is an emerging consensus within veterinary medicine on the need to understand senile dementia in our pet population (7–11). fCDS is currently a diagnosis of exclusion and like human counterparts; there are limited treatment options. In a seminal paper published in 2007, Dr. Gunn-Moore and Dr. Head review the lab evidence, behavioral changes and neuropathology unique to fCDS in geriatric cats (12). Recent studies confirm and extend the human-like neuropathology phenotype as ever closer to many human NDD (13, 14). Much like humans in early stages of NDD, the initial clinical feline characteristics present as *disorientation, short-term memory loss, sleep/wake disturbances, incontinence, anxiety and increased vocalizations* (15).

The unfolded protein response (UPR) is a highly conserved evolutionary pathway. Within the endoplasmic reticulum (ER), nascent polypeptide chains are synthesized on associated ribosomes or imported for post-translational modifications, and further folded into cellular proteins to be exported through Golgi-mediated pathways. Imbalances in this “conveyor belt” system cause backups and accumulation of defective or misfolded proteins, in essence blocking the carefully refined proteostasis between synthesis and degradation of cellular proteins; reviewed in (16–22). When the tipping point between accumulation and ER degradation of defective proteins in the ER lumen occurs, a condition called “ER stress” results. Resolving ER stress is critical to cell survival. Eukaryotic cells have evolved three distinct signaling pathways (UPR) to reprogram protein expression at multiple levels. Gene expression is regulated by activating transcription factor 6 (ATF6). Protein synthesis is regulated by eukaryotic initiation factor 2 (eIF2α) kinase (PERK), and modulation of protein folding through type I transmembrane protein inositol requiring 1 (IRE1α). These pathways are linked to ER-associated protein degradation (ERAD) and lysosomal degradation to remove problematic proteins (16).

Mild ER stress from temporary or mild physiological or pathological events is resolved through adaptive cytoprotective UPR mechanisms. However, continuous, or excessive protein synthesis of imperfect product or a slowing of the transport or degrading systems induces prolonged activation of UPR pathways, leading to maladaptive cellular signaling and ultimately, cell damage or death. Given the abundance of potential aggregating proteins associated with NDD, it is not surprising that links between ER stress and upregulation of UPR mechanisms is an emerging therapeutic area for all forms of NDD (18, 23–26).

Another critical factor upregulating maladaptive UPR pathways is the presence of neuroinflammation. Activated UPR components have been shown to increase known pro-inflammatory cytokines, IL-6, TNF-α, IL-1β, through the common transcription factor nuclear factor-κβ (NF-κβ) (27). Within the CNS, both microglia and astrocytes can synthesize and secrete pro-inflammatory proteins and cytokines, beginning a positive feedback loop and crosstalk between increasing ER stress, upregulation of UPR mechanisms and increasing levels of neuroinflammation. This prolonged activation of pro-apoptotic components of the UPR eventually leads to synaptic loss or neuronal cell death (28). Aβ accumulation may be an early triggering mechanism for this crosstalk loop initiation. When Aβ is administered to rats, an upregulation of ER stress and inflammatory markers is seen with a concomitant increase in cognitive impairment (29).

Oxidative stress is the third branch of a triumvirate with UPR components and neuroinflammation in the pathway to NDD. This state is an imbalance between reactive oxygen species (ROS) and antioxidant mediators (redox). Outside of mitochondrial oxidative phosphorylation, the ER generates about a quarter of total cellular ROS through protein folding (30). Disulfide bridging, required for some protein folding, occurs in the oxidizing environment of the ER lumen. Problems arise when oxidative stress overwhelms ER ROS defenses and protein transport is impeded (31). The UPR is then activated and if the excess ROS becomes chronic, neuronal apoptosis is likely (32).

Cats are unique pets. Dogs have lost genetic components found in their wild counterparts, allowing for easier cohabitation with humans (33). Paleogenetic studies support the concept that cats, not humans, initiated their coexistence (34, 35). Modern feline DNA is nearly identical between the suspected wild ancestor of our modern housecat; *Felis sylvestris lybica* and current pet or feral felines *(Felis sylvestris catus).* However, key genetic signatures have been found in specific genes associated with behavior and reward systems (36) in cats living with humans, demonstrating the physical and social pressures cats may experience when dwelling amongst humans. Indoor only cats display enhanced problem-solving in a task that assesses social engagement with humans over mainly outdoor living cats (37). Actual feline facial morphology and expressions have been shaped by interactions with humans (38). Humans apparently like their housecats to appear “cute” over feral. Cats may be able to somewhat adapt to our world but the stressors can occasionally become overwhelming (39), with predictable behavioral issues.

**Figure 1:**
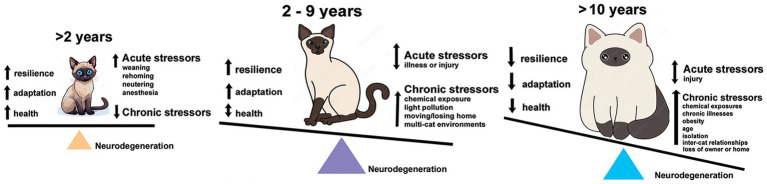
Tipping the scales over the lifespan of an owned cat towards neurodegeneration. Kittens and adolescent cats have capacity for resilience and adaptability to acute stresses and have fewer interactions with chronic stressors. By the time they reach adulthood, the burden of chronic stressors is accelerating and neural damage accumulating. Senior cats (10 years and older) are exposed to a multitude of stresses and increasing effects of health and age-related issues just as humans. Their capacity for resilience and adaptation is more limited, further increasing the potential for neural damage and loss.

There are few comparative studies of stressors between human and felines (40, 41). Funding for companion animals, cats in particular, is limited for pathologies such as fCDS. Given those constraints, chronic stressors must be viewed synergistically between humans and cats. Common environmental stressors are shared within a household and affect each species simultaneously (42). Many studies exist on effects of stressors on humans or model systems, like mice, rats, or dogs, but far fewer exist for felines alone. This review will breakdown what is known about the effects of predictable stressors on cats, categorized as *environmental*, *social-behavioral*, and *physiological* stress, and the potential for oxidative stress, neuroinflammation or increased expression of activated UPR from these stressors to induce cellular damage or cell death leading to an NDD-like pathology (Figure 1).

### Experimentally known effects of stressors on domestic cats

#### Environmental stressors

Although this review will mainly focus on research using client-owned cats, rather than feral individuals, many of the same environmental conditions exist for free-roaming or feral animals. Toxic chemicals, noises and excessive lighting exist as abundantly in outdoor environments as in our home settings. One of the most prominent indoor pollutants is tobacco smoke and the associated toxins from either cigarettes or vaping. Cats have highly developed olfactory systems, thus, even small amounts of second or third hand smoke evoke biochemical responses. Like human counterparts, cats respond to tobacco smoke with increased levels of pro-inflammatory cytokines, indicating states of enhanced oxidative stress (43). As neurons require significant oxygen to maintain high levels of oxidative phosphorylation, an imbalance between reactive oxygen species (ROS) and antioxidant compounds exists. Microglial phenotypes are found to be altered in human cell cultures following exposure to nicotine, further increasing localized ROS (44). The ER has a critical function in oxygen regulation and in situations of oxidative stress, the UPR is upregulated to mitigate the damage. Prolonged exposure to cigarette smoke for cats will therefore be considered a chronic stressor, with the potential to trigger the apoptotic wing of UPR over time in neurons by the generation of pro-inflammatory cytokines and increasing levels of oxidative stress in microglia and astrocytes.

Cats develop acute hearing at very early ages. They can discriminate ambient sounds outside of the human auditory range. Computer monitors, fluorescent light bulbs and other high frequency emitters are within their capabilities. Loud unpredictable sounds cause indoor cats stress when hiding places are limited. Resident and ambient noise is a consistent feature in homes. A typical range has been measured at 51–78.2 dB (45). White noise in the range of 5-20 kHz at 115 dB leads to auditory neuron degeneration in mice within hours of exposure (46). The same range of exposure in adult male cats resulted in synaptic dysfunction within the colliculus inferior over a period of 10 days exposure to 1 hour of noise daily (47). Whether synaptic dysfunction proceeds or precedes oxidative stress, the result is the same; neuronal loss and overall neurodegeneration (48). Thus, our noisy lives, with music, TV, traffic background and electronic white noise, has the potential to increase ROS, and begin the downward spiral of oxidative stress-UPR-neuroinflammation cycles.

Humans have disrupted the natural cycles of light and dark with artificial lighting. We have even stretched that further with bright LED illumination, increasing blue light (λ = 460–480 nm) and electronics left on 24 h. Light cycles are a critical aspect of circadian rhythms that govern wakefulness, sleep pattern and daily functioning. All cats sleep a typical 12–20 h per day, with peak wakefulness at sunrise or sunset to coordinate with prey activity. Light can affect the sleep of domestic cats, as it does in humans or rodents, by depressing expression of melatonin, interrupting normal intensity of sleep patterns or extend diurnal or nocturnal activity (49). Nighttime light exposure is shown to increase depression in humans (50, 51) and in rodent models (52–54). An unexplored aspect of sleep disruption is a recently recognized impairment of the brain waste disposal system; the astrocyte-mediated glymphatic system (55). Sleep disturbances increase CSF levels of toxic Aβ (56, 57), which accumulates into extracellular plaques. Hyperphosphorylated tau is usually found intracellularly but a recent study has found extracellular tau in the glymphatic system, indicating clearance could also be impaired with altered sleep (58). As cats present with both senile Aβ plaques and NFT tau isoforms (1, 3–6), stress of nighttime light may contribute to developing NDD.

Further increasing stress in a domestic cat are human expectations. Humans typically sleep between 10 PM – 6 AM and dislike nighttime disturbances such as wandering or pouncing cats. Playtimes are shifted towards daytime when cats would naturally be inactive. Domestic cats have had to suppress typical behaviors to maintain their human-cat bonds. Therefore, their ingrained sleep patterns may become more fragmented. In mice, fragmented sleep increases Aβ (40 and 42) in the hippocampus and increases microglial activation and secretion of pro-inflammatory cytokines (59). Dim light exposure of pig retinal organotypic cultures up-regulates markers of ER stress and electron microscopic analysis reveals photoreceptor degeneration (60). Our continual use of artificial light may be a major triggering mechanism over a lifespan of a cat towards NDD.

#### Social - behavioral stressors

Traditionally considered solitary hunters (61), modern feral cats frequently reside in large colonies (62). Although, there is limited research on domestic cats and social structures (63, 64), studies exist on larger cat species from zoos suggesting housing with unrelated conspecifics, and a lack of hiding spaces contributes to captive large cat stress (65, 66). Within a household, solitary cats do not have the daily stress of feline hierarchical conflicts, but in multi-cat homes, such daily stressors can have a profound effect on individual cats. A 2020 survey found that almost 74% of owners reported inter-cat conflict signals early in stages of introduction, with 50% reporting physical contact. There was a correlation between the number of resident cats and the reporting of conflicts (67). Some earlier studies show conflicting data. Urinary or fecal cortisol levels were not significantly different in multi-cat, single cat or sheltering housing conditions (68, 69), indicating that circulating cortisol levels do not fully reflect the full spectrum of behavioral aspects of feline stress. A more realistic assessment for feline stress relies on observational scores for behavioral demeanor for individual cats (70).

Individual cats frequently respond differently to stimuli regardless of their social ranking (71). Cats can and do live cooperatively. Yet, many feel and react to the stress of a lack of hiding resources, limited litter resources and especially the presence of a dominant bully cat. The aggressor cat and the bullied cat are at risk for oxidative stress and neuroinflammation (72). When aggressive mice are treated with antioxidants, biomarkers for oxidative stress, pro-inflammatory cytokines and aggressive behaviors decreased (73). The less dominant cat is at risk for depression, just as their human counterparts (74–76). Major depressive disorder (MDD) has its pathological roots in dysfunctional neurotransmission. Yet, there is increasing evidence that MDD further triggers neuroinflammation and concomitantly induces ER stress and upregulation of activated UPR components (77–79).

More pet parents are bowing to increasing public pressure to protect cat’s lives by restricting or eliminating their access to the outdoors. Although their impacts to wildlife are overblown (80), the dangers to owned pets by cars, coyotes, theft, or injury is real. For many cats, restricting their movements is a real stressor (81). In rodent models of restraint, rats display depressive like behaviors; reviewed by (82) and have increased expression of UPR components (83). Living in close contact with humans creates social stresses in our territorial pet cats that earlier generations of felines never experienced. Multi-cat homes, less range, and more restrictions over fundamental physiological needs has provided a seeding ground for psychological issues that could progress to actual neurological pathologies.

#### Physiological stressors

A major human risk factor for development of NDD is obesity (84, 85). Obesity is not a typical problem for feral cats, as they rely on prey availability. The story is quite different for owned domestic cats. Worldwide, an estimated 11–63% of household living cats are overweight (86) and a third considered obese. Extra weight is a “heavy” physiological stressor, and for cats, a direct consequence of human control over nutritional choices for their cats. Hematological oxidative stress markers are elevated in obese cats over control and overweight cats (87), predisposing animals to neuroinflammation. Compelling evidence from human obesity studies, mainly using advanced imaging techniques, demonstrate significant structural changes in brains of obese individuals (88–91), with atrophy of key areas such as the hippocampus and subcortical structures. Hand in hand with obesity, is the specter of physiological malnutrition of domestic cats. As obligate carnivores, commercial foods high in fat, consisting of unnatural plant-derived proteins, and carbohydrate additives, such as corn, soy or grains, combined with low levels of omega-3 and omega-6 polyunsaturated fatty acids (PUFA), are not adequate substitutes for pure animal proteins (92). Insufficient intake of animal-derived essential amino acids (taurine for cats), vitamin D3 and cholesterol can impede neuronal development or effect feline cognition (93, 94). Examples of diet-related cognitive dysfunction are found in other animal models. Thiamine deficiency (vitamin B1) increases oxidative stress and is associated with neuroinflammation leading to potentially irreversible hippocampal damage in an organotypic culture experiment (95). Oxidative stress and impaired spatial memory are a feature of a mouse model of protein malnutrition (96). Combining overfeeding our cats, and the physiological stress of a nutrient restrictive diet, both of which have the potential to upregulate maladaptive UPR mechanisms, humans are creating conditions appropriate for development of NDD feedback loops.

Age is cited as the most prominent risk factor in human NDD. It is a stressor that both humans and cats share. We have expanded human life with advanced medicine. Bringing our cats inside and providing steady diets, effective vaccinations and life-saving care has greatly extended previous definitions of feline lifespans. It is not usual for owned cats to live well beyond 16 years, while feral cats struggle to <5 years. With this increase in life expectancy comes a staggering increase in the incidence of feline cognitive dysfunction (fCDS) (11, 14, 15, 97–99). Extrapolating from human studies of aging and neuroinflammation, cats are expected to display similar increasing markers of neuroinflammation (100), but direct studies are lacking. Aged brains have increased levels of oxidized proteins and changes in membrane fatty acid composition with increased amounts of ROS susceptible monosaturated fatty acids and arachidonic acid (AA) (101). Systemic oxidative stress is reported in aging cats, with male cats at greater risks (102). Currently, there is more research on canine brain health than cats.

## Discussion

Finding a translational animal model where early dementia-like behaviors and correlative biochemical signatures can predict final cognitive decline is a big leap forward in our understanding of the etiology of NDD. Homed or sheltered cats may fill this current void, benefitting cats and humans. That we have this ideal model sitting in our living rooms today, makes this idea even more novel and exciting. Human-influenced stressors in cat’s lives have the potential to prime the cycle of neuronal damage, synaptic dysfunction, and maladaptive glial function, all leading up to neuron death and degeneration. Prevalence of fCDS is on the rise as our cat population ages. Whereas canine CDS is well defined, feline CDS remains mysterious due to lack of awareness by owners and veterinarians and the ability of cats to hide symptoms. Like human NDD, there are no effective pharmacological interventions to stop the neurodegeneration once it begins. Thus, understanding potential triggers of neuronal damage may hold keys to identifying at risk feline populations and finding efficacious treatments. Triggers may be multi-factorial, combining throughout the life of a cat with the endpoint being NDD. With a shorter course of disease, assessing therapies that control cognitive loss and brain loss become attainable goals. Importantly, the ability to find the earliest biochemical signatures of disease becomes possible. At risk cats, individuals with a combination of identified life stresses along with careful owner observation, can be tracked over time looking for changes in biochemistry and behaviors that eventually overlap with fCDS signalment (98) [Basepaws Aging survey]^1^. Thus, we advocate for more funding and more translational grants for feline research. Why else would the National Center for Advancing Translational Sciences be called NCATS?

## Author contributions

IN: Conceptualization, Investigation, Writing – original draft, Writing – review & editing.

## Footnotes

^1^Feline Longevity Research at Basepaws.
